# Switching Virally Suppressed, Treatment-Experienced Patients to a Raltegravir-Containing Regimen Does Not Alter Levels of HIV-1 DNA

**DOI:** 10.1371/journal.pone.0031990

**Published:** 2012-03-01

**Authors:** Yu Ming Paul Lam, Kristin L. McBride, Janaki Amin, Damien V. Cordery, Anthony D. Kelleher, David A. Cooper, Kersten K. Koelsch

**Affiliations:** 1 The Kirby Institute, University of New South Wales, Sydney, Australia; 2 St Vincent's Hospital Sydney, Centre for Applied Medical Research, Darlinghurst, New South Wales, Australia; 3 St Vincent's Hospital Sydney, Darlinghurst, New South Wales, Australia; University of Toronto, Canada

## Abstract

**Background:**

Current HIV-1 antiretroviral therapy (ART) greatly reduces virus replication but does not significantly affect the viral reservoir. Raltegravir, a recently introduced integrase inhibitor, could, at least theoretically, reduce residual viremia in patients on ART and affect the viral reservoir size. The aim of this study was to assess whether switching therapy in treatment-experienced patients that were virally suppressed to a raltegravir-containing regimen reduces the size of the viral reservoir, and if such treatment leads to a change in levels of HIV 2-LTR circles in this patient group.

**Methods:**

14 ART experienced individuals with a suppressed viral load (<50 HIV-1 RNA copies/mL plasma) at baseline (for at least 2 months) were switched to a raltegravir-containing regimen. Blood samples were taken at baseline and at ≥2 timepoints up to 48±6 weeks. Levels of total HIV-1 DNA and 2-LTR circles in peripheral blood mononuclear cells (PBMCs) were measured using real-time PCR assays.

**Results:**

There was no significant change in HIV-1 total DNA levels over the study duration (p = 0.808), median slope 0.24 (conservative nonparametric 95% CI: −11.78, 26.23). Low levels of 2-LTR circles were detected in 2 patients. One had 16 copies/10^6^ PBMCs at baseline and the other had 34 copies/10^6^ PBMCs at week 51.

**Conclusions:**

The switch to a raltegravir containing regimen was not associated with a significant change in HIV-1 total DNA levels in this cohort. There were no observed changes in the levels of HIV-1 2-LTR circles associated with raltegravir treatment initiation.

## Introduction

The human immunodeficiency virus type 1 (HIV-1) viral reservoir is established early in infection [1] and is the major barrier to virus eradication and thus a cure for HIV-1 infection. [2], [3], [4]. Several mechanisms may contribute to the maintenance of the viral reservoir including the homeostatic dynamics within the CD4+ T cell pool, anatomical sanctuary sites as well as low level ongoing replication and consequently new rounds of virus infection. If ongoing replication despite seemingly suppressive ART was a significant contributor for the maintenance of the HIV-1 reservoir size, then intensification with more potent agents or regimens may be able to further reduce levels of HIV-1 DNA in peripheral blood of the treated individuals. In support of ongoing replication, evidence of cross infection has been demonstrated between resting and activated CD4+ T cell compartments [5] and between GALT and peripheral blood mononuclear cells (PBMCs) [6] in patients on suppressive ART.

Raltegravir is an integrase inhibitor which has been shown to significantly alter viral decay dynamics in treatment naive patients [7], and which is also very effective in treatment experienced patients [8]. In treatment experienced but virally suppressed patients raltegravir could, at least theoretically, further inhibit the HIV-1 life cycle by preventing integration of persisting linear non-integrated HIV-1 DNA through the following mechanisms: direct inhibition via its mechanism of action, increased pharmacological penetrance into sanctuary sites compared to other antiretroviral drugs [9], and/or by inhibiting linear non-integrated HIV-1 DNA that had escaped only partially inhibitory upstream NRTI or NNRTI therapy, for example from drug resistance mutations. We hypothesized that raltegravir could affect the size of the HIV-1 reservoir by preventing its maintenance via the mechanisms listed above. In the presence of ongoing replication, integrase inhibitors may also increase the levels of episomal, 2-LTR circle DNA. 2-LTR circle increases have been detected after integrase inhibitor treatment in cell culture [10], animal models [11], and in patients [12].

At the time of study conception, there was no information on the effects of raltegravir containing ART regimens on HIV-1 DNA levels. We hypothesized that in a treatment experienced cohort, including patients on salvage therapy regimens with potentially higher levels of residual viraemia, maintenance of the viral reservoir through ongoing replication might be more prevalent. Switching to a raltegravir containing regimen may therefore further suppress ongoing replication in these patients and have an impact on the size of the viral reservoir. We assessed total HIV-1 DNA levels in PBMC as a measure of the viral reservoir from 14 treatment experienced patients with suppressed viral load (<50 HIV-1 RNA copies/mL plasma) at study entry that were switched from their current ART to raltegravir containing regimens. We also assessed levels of HIV-1 2-LTR circles since changes in levels of episomal DNA after therapy switch would be an indicator for the presence of ongoing replication in this cohort.

## Methods

### Ethics statement

Written informed consent was obtained from all patients who participated in this study. The study was approved by the human research ethics committee, St Vincent's Hospital, Sydney, Australia (file number 08/SVH/58).

### Participants

14 treatment experienced patients with suppressed viral load (<50 HIV-1 RNA copies/mL plasma) at least two months prior to study entry were switched from current ART to raltegravir containing regimens. The decision to switch therapy was primarily based on the treating physician's attempt to achieve an optimised treatment regime. Viral load was assessed using the standard Roche COBAS Amplicor assay version 1.5 (Roche Diagnostics, Pleasanton, USA). Blood samples were taken at baseline and at approximately twelve-week intervals, with 2 or more subsequent samples for each patient up to 48±6 weeks.

### DNA extraction

PBMCs were extracted from blood samples with Ficoll-Paque gradient density centrifugation [13] and enumerated. DNA from 5–10 million PBMCs was extracted with the DNeasy Blood & Tissue Kit (Qiagen Kit No 69504, Doncaster, Australia) according to the manufacturer's recommended protocol with some modifications: 1. The cell lysis step was extended to 16 hours. 2. The DNA elution steps were extended to 1 hour each. 3. The AE elution buffer was replaced with Tris-hydrochloride (Tris-HCL) (10 mM, pH 8.0) (Invitrogen). The elution step was repeated to obtain maximum yield. Extracted DNA was stored at 4°C.

### Total HIV-1 DNA quantification with real-time PCR

HIV-1 DNA was quantified using a real-time PCR assay targeting a highly conserved 155 base pair region of the *gag* gene as previously described [14]. In brief, primers and probes used were:

HIV-gag sense primer SK145: 5′- AGTGGGGGGACATCAAGCAGCCATGCAAAT-3′

HIV-gag antisense primer SKCC1B: 5′- TACTAGTAGTTCCTGCTATGTCACTTCC-3′

Fluorescent locked nucleic acid (LNA) probe SKLNA2–3:

5′-6-FAM-AT[C]A[A]T[G]AGGAA[G]CT[G]C-TAMRA-3′

Bases in brackets indicate locked nucleic acids (LNAs).

Patient samples and standard curve dilutions were run in duplicate in 25 µl reactions with 12.5 µl of iQ Supermix (Bio-Rad Laboratories, California, USA), 800 nM of SK145 and SKCC1B, and 200 nM of SKLNA2–3. A maximum input of 500 ng DNA (approximately 80,000 cells) input was used per reaction. DNA from one HIV-1+ individual with known HIV-1 DNA copy numbers was used as a reference control. The standard curve was designed using linearised HIV-1 plasmid pNL4-3 in 7 serial 10-fold dilutions from 10^7^ to 10^1^ copies. The limit of detection for the assay was 10 copies. Cycling conditions for both assays were: 1 cycle −95°C for 3 minutes and 40 cycles −95°C for 15 seconds, 60°C for 1 minute on a Bio-Rad iQ-5 multicolour real-time PCR machine (Bio-Rad Laboratories).

HIV-1 total DNA copy numbers were normalised with a TaqMan beta-actin assay (Applied Biosystems, California, USA) to determine copies per 500 ng genomic DNA input (under the assumption that 8×10^4^ cells = 500 ng of DNA), this was then multiplied by 12.5 and displayed as copies per 10^6^ PBMCs. All samples from the same patient (baseline to 48±6 weeks) were run within the same PCR plate to enable comparison between timepoints without the need to account for run-to-run variation. All assays used non-template negative controls.

### 2-LTR circle quantification with real-time PCR

2-LTR circles were quantified using a real-time PCR assay targeting a 297 base pair region of the 2-LTR junction. Primers and probes used were:

Sense primer 2-LTR JF: 5′-GCTAACTAGGGAACCCACTGCTTAAG-3′

Antisense primer HIV R: 5′-ACTGGTACTAGCTTGTAGCACCATCCA-3′

LNA probe Mf374:

5′-6-FAM-ACA[C]A[C]A[A]G[G][C]T-TAMRA-3′

Patient samples were run in triplicate in 25 µl reactions with 12.5 µl of iQ Supermix, 280 nM of sense and antisense primers, and 200 nM of Mf374. A maximum input of 500 ng DNA input was used per reaction. Cycling conditions were as described in the above assay with the second step increased to 45 cycles. Criteria used to define a positive result were ≥2 of 3 wells with a threshold cycle before or within 1 cycle of the 10^1^ standard. An in house HIV-1+ patient sample was used as a positive control. The standard curve was designed using extracted plasmid DNA containing the 2-LTR junction region in 7 serial 10-fold dilutions from 10^7^ to 10^1^ copies. The limit of detection for the assay was 10 copies. 2-LTR circle copy numbers were normalised with a TaqMan beta-actin assay as described above.

### Statistical analysis

The HIV-1 total DNA results of each patient were graphed against time. A linear trendline was plotted for each patient and the slopes of the trendlines were calculated. The nonparametric Wilcoxon Sign Rank test was used to evaluate if the median slope significantly deviated from 0 (null hypothesis). The patients were further sub-grouped by time of virological suppression prior to baseline (<12 mths and ≥12 mths) and by reverse transcriptase inhibitors in the treatment regimen. The Wilcoxon Sign Rank test was then used to assess if the median slopes within these subgroups deviated from 0. Again, a median trendline slope of 0 (null hypothesis) would indicate no change in the HIV-1 DNA levels over the study duration. All statistical analysis was performed using GraphPad Prism version 5.0 c (GraphPad Software, Inc).

## Results

14 treatment experienced patients with suppressed viral load (<50 HIV-1 RNA copies/mL plasma) at study entry were switched to raltegravir containing regimens. The patients in the study cohort were all male with a median age of 54.0 years (range 31.9–74.0). Table 1 shows the ART regimens of the study cohort before and after the switch to raltegravir and the duration of virologic suppression (<50 HIV-1 RNA copies/mL plasma) before the switch (median: 21 mths, range: 2–60). All patients remained virologically suppressed at all time points throughout the study.

**Table 1:** Study cohort characteristics and slope of HIV-1 Total DNA trendlines.

Patient ID	Previous regimen	Switch regimen	Virologic suppression (months)	HIV-1 DNA level (copies/10^6^ PBMCs)		Slope of trendline*
				Baseline	Endpoint	
001	FOS, TDF, FTC	RAL, TDF, FTC	33	1587	4124	49.2
002	FOS, TDF, FTC	RAL, TDF, FTC	7	7643	3154	−85.8
011	LPV/r, ABC, 3TC	RAL, ATV	57	2212	3867	26.2
012	LPV/r, ABC, 3TC	RAL, TDF, FTC	2	4580	8105	229
013	EFV, FOS, LPV/r	RAL, DRV/r	47	291	790	10.7
014	ATV/r, TDF, FTC	RAL, ATV	24	1528	904	−13.7
017	ATV/r, TDF, FTC	RAL, ATV	11	579	116	−6.89
018	DRV/r, TDF, FTC	RAL, ATV, TDF, FTC	14	5824	4883	−11.8
021	T20, DRV/r, TDF, FTC	RAL, DRV/r, TDF, FTC	11	1281	1571	4.82
022	SQV, LPV/r	RAL, DRV/r	60	381	643	7.01
023	AZT/3TC, DRV/r	RAL, DRV/r	18	1456	1091	−11.6
026	NVP, ABC, 3TC	RAL, ATV	54	946	1462	10.1
027	SQV, ABC, 3TC, LPV/r	RAL, DRV, 3TC	16	482	281	−4.33
034	ABC, 3TC, SQV/r, ATV	RAL, ATV	31	325	114	−6.56

Note: All patients had HIV-1 RNA levels of <50 copies per mL of plasma for the duration of the study. *: P-value not significant for all slopes. 3TC, lamivudine; ABC, abacavir; ATV/r, ritonavir-boosted atazanavir; AZT, zidovudine; DRV/r, ritonavir-boosted darunavir; EFV, efavirenz; FOS, fosamprenavir; FTC, emtricitabine; LPV/r, ritonavir-boosted lopinavir; NVP, nevirapine; RAL, raltegravir; SQV, saquinavir; T20, enfuvirtide; TDF, tenofovir.

### HIV-1 Total DNA

**Figure 1:**
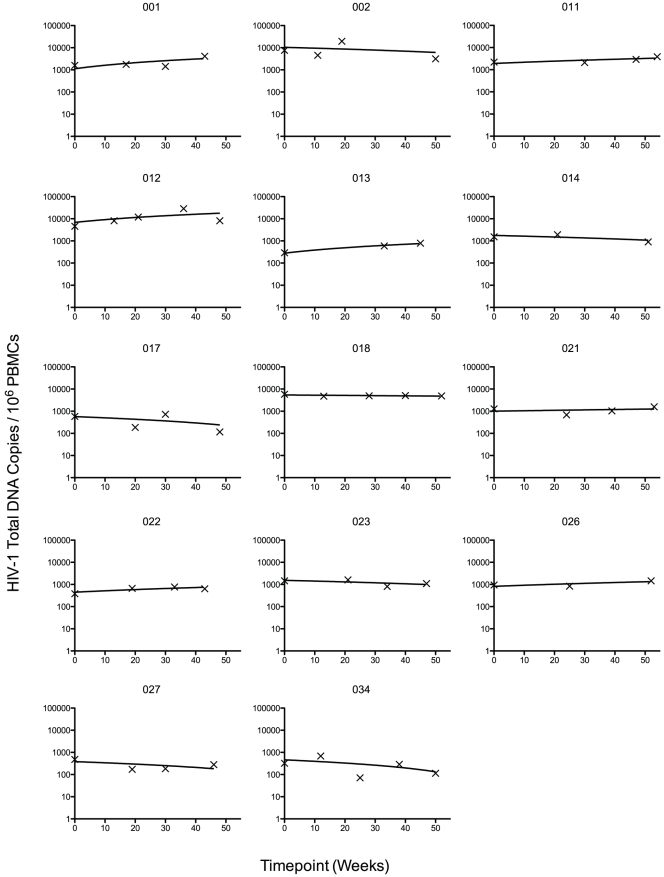
Plot of HIV-1 total DNA results for individual patients. Plots of the 14 study patients: Each plot depicts an individual patient's levels total HIV-1 DNA levels in PBMCs (black ‘X’es) from baseline (week 0) to study endpoint. Solid lines are linear trendlines and may appear curved due to the log scale of Y-axis. The linear trendline demonstrates if the level of HIV-1 total DNA increased or decreased in each patient after the switch to a raltegravir-containing regimen.

**Figure 2:**
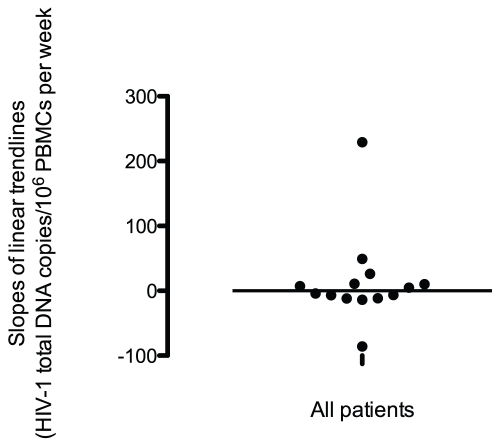
Slopes of HIV-1 total DNA trendlines for all patients. The slopes of the linear trendlines for the study cohort are shown here. The horizontal solid line indicates the median trendline slope. A nonparametric one-sample Wilcoxon Sign Rank test of the slopes demonstrated no significant difference from 0 (p = 0.808), indicating no change in the total DNA levels of the cohort over the study duration.

HIV-1 total DNA was detectable in all patient samples. Figure 1 shows the levels of HIV-1 total DNA/10^6^ PBMCs for each patient (range: 71.3–28,564 copies/10^6^ PBMCs). A linear trendline was plotted for each patient, and the slope of each trendline was obtained (Table 1). Figure 2 shows the slopes of the linear trendlines for all patients (range: −85.8–229.2 copies/10^6^ PBMCs per week). A nonparametric one-sample Wilcoxon Sign Rank test of the linear trendline slopes found no significant difference from 0 (p = 0.808) and a median slope of 0.24 (conservative nonparametric 95% CI: −11.78, 26.23), indicating no change in the total DNA levels over the study duration.

**Figure 3:**
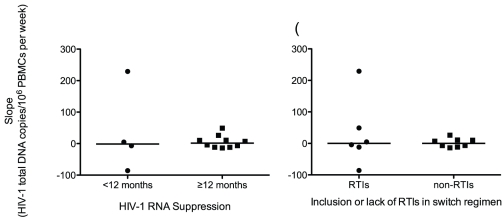
Slopes of HIV-1 total DNA trendlines grouped by A) duration of virologic suppression prior to switch, and B) RTIs in switch regimen. Panel A) The study cohort was grouped into <12 months (n = 4) and ≥12 months (n = 10) of virologic suppression prior to the switch to a raltegravir-containing regimen. Analysis of the slopes of trendlines showed no significant difference from 0 in either group (p = 1.00 in <12 months and p = 0.770 for ≥12 months), indicating no effect of length of suppression prior to switching on the attenuation of total HIV-1 DNA levels. Panel B) The study cohort was grouped by the inclusion (n = 6) or lack of (n = 8) RTIs in the switch regimen. Analysis of the slopes of trendlines showed no significant difference from 0 in either group (p = 0.844 in both groups), indicating no measurable effect of the inclusion or lack of RTIs in our study cohort.

When the study cohort was subdivided into patients with <12 months (n = 4) and ≥12 months (n = 10) of virologic suppression prior to the switch to raltegravir (Figure 3A), analysis of the slopes of trendlines in both groups demonstrated no significant difference from 0 in either group (p = 1.00 in <12 months and p = 0.770 for ≥12 months). In a separate analysis, the study cohort was grouped by inclusion (n = 6) or lack of (n = 8) reverse transcriptase inhibitors (RTIs) in the treatment regimen after the switch (Figure 3B). Neither group had a median trendline that was significantly different from 0 (p = 0.844 in both groups).

### 2-LTR circles

**Figure 4:**
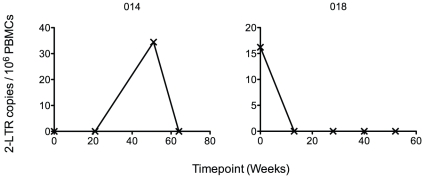
HIV-1 2-LTR assay results. Low levels of 2-LTR circles were detected at week 51 in patient 014 and at baseline in patient 018. 2-LTR circles were not detected at any timepoint for the remaining 12 of 14 patients in the study.

Two patients had detectable 2-LTR circles at one timepoint each. Patient 018 had 16 copies/10^6^ PBMCs at baseline and Patient 014 had 34 copies/10^6^ PBMCs at week 51 (Figure 4). No 2-LTR circles were detected in 12 of 14 patients. 2-LTR circles were therefore not detected in 54 out of 56 total timepoints in the study.

## Discussion

This study assessed the impact of raltegravir on the levels of total HIV-1 DNA and 2-LTR circles in 14 treatment experienced patients with suppressed plasma viraemia before switching therapy. Our results clearly demonstrate that a switch to a raltegravir containing regimen in patients on stable ART was not associated with a decay in HIV-1 total DNA over 48±6 weeks. In the setting of low-level residual viral replication, treatment intensification or switching to a more potent regimen might be expected to result in decay of the viral reservoir if ongoing replication was essential to maintenance of the reservoir size. Although the potency of raltegravir containing regimens remains to be fully established, we did not observe any virological failure during the first 48 weeks after switch of therapy, and also during an extended observation of this cohort over 96 weeks (data not shown). This includes those patients that were switched to a simplified raltegravir/atazanavir regimen. Similar results were reported in a recent study by Cordery et al. [15]. The stability of HIV-1 DNA as demonstrated in our study therefore indicates that inclusion of raltegravir to the treatment regimens did not significantly alter the size of the viral reservoir.

These findings are in line with other recent studies [12], [16], [17]. It is worth noting, that the clinical significance of changes in HIV-1 DNA levels in peripheral blood remains to be fully elucidated. Yukl et al. [18] have recently demonstrated that the amount of HIV-1 DNA in CD4+ T cells is on average 5–10 fold higher in the gut compared to peripheral blood, suggesting that the reservoir size in the gut may exceed previous estimates of the total HIV reservoir size by an order of magnitude. In another study, Chun et al. [19] reported rebound of plasma viraemia following ART interruption in an individual with undetectable HIV-1 DNA in CD4+ T cells as well as GALT, and a profoundly low level of replication competent virus, indicating that viral rebound may not be indefinitely suppressed even in a patient with virtually undetectable HIV DNA levels. Along with other studies [12], [16], [17], our results show that the simple inclusion or addition of an integrase inhibitor like raltegravir into an ART regimen is unlikely to significantly reduce the size of the viral reservoir in ART treated patients who already have suppressed plasma viral loads. The clinical relevance of stability or changes in HIV-1 DNA levels will require further exploration in future studies, and additional markers of the viral reservoir size might be necessary to better predict clinical outcome.

The persistence of ongoing rounds of viral suppression during ART is currently a matter of debate. Findings that suggest ongoing replication include the detection of HIV-1 RNA in lymphoid and gut tissue as well as evidence of cross-infection between cellular compartments in CD4+ T cells and GALT, despite undetectable HIV-1 RNA in plasma [5], [6], [20], [21], [22]. However, recent pharmacodynamic modeling indicates that current ART can fully inhibit ongoing cycles of replication [23], and several other recent studies also provide evidence against ongoing replication as a significant mechanism through which the viral reservoir is maintained [24], [25], [26], [27]. We did not measure ultra low-level viraemia and therefore cannot assess if raltegravir had an effect on residual HIV-1 RNA levels independent of the total DNA results. However this is unlikely since other studies have shown no change in low-level viraemia with switching to raltegravir [28] or intensification of treatment with raltegravir [27], [29].

Low levels of 2-LTR circles, a possible surrogate marker of ongoing replication during raltegravir treatment [12], were detected in only 2 of 14 patients in our cohort. Increases in 2-LTR circle levels have been shown to be associated with integrase inhibitor activity in the presence of viral replication in vitro [30], [31] and in vivo [12]. Our findings of no significant change in 2-LTR circle levels after switching to raltegravir, together with the lack of change in HIV-1 total DNA levels, most likely reflect only minimal or no ongoing replication in this cohort. Again, our results are in line with findings from Delaugerre et al. [16], who also did not detect increases in 2-LTR circles 24 and 48 weeks after the switch to a raltegravir-containing regimen in treatment experienced patients. In another study, Buzon et al. [12] identified increases in 2-LTR circles at 2 and 4 weeks after treatment intensification with raltegravir in virologically suppressed patients, mainly in those who had been on a protease inhibitor containing regimen. Two other studies in treatment naive patients [32], [33] indicate that peak changes in 2-LTR circles occur within the first 12 weeks after treatment initiation with or without the inclusion of an integrase inhibitor in the treatment regimen. One limitation of our study was that the earliest follow-up measurement was more than 12 weeks after baseline for many patients, and any early increases in 2-LTR circles might therefore have been missed. In addition, quantitative assays for measuring 2-LTR circles are also technically demanding due to the high sequence variability of the 2-LTR junction region [30], so the lack of detectable changes in 2-LTR levels might be, at least in part, a result of these limitations.

In summary, our results demonstrate that the switch to a raltegravir containing regimen in a cohort of treatment experienced, virologically suppressed patients was not associated with a decay in HIV-1 total DNA over 48±6 weeks, and indicates that this change in treatment regimen did not have a significant impact on the viral reservoir size or mechanisms of maintenance. The inclusion, or as other groups have shown the addition of raltegravir to an ART regimen is thus unlikely to contribute significantly to reservoir clearance without other interventions. Future studies exploring therapeutic approaches to eradication of HIV-1 infection will likely need to attempt to specifically target latently infected cells in addition to the complete reduction of all ongoing replication. Finally, the clinical relevance of changes of cell associated HIV-1 DNA levels in the setting of therapeutic strategies that aim at reducing the HIV reservoir will also need to be more clearly established.

## References

[1] Chun TW, Engel D, Berrey MM, Shea T, Corey L (1998). Early establishment of a pool of latently infected, resting CD4(+) T cells during primary HIV-1 infection.. Proceedings of the National Academy of Sciences of the United States of America.

[2] Chun TW, Carruth L, Finzi D, Shen X, DiGiuseppe JA (1997). Quantification of latent tissue reservoirs and total body viral load in HIV-1 infection.. Nature.

[3] Wong JK, Hezareh M, Gunthard HF, Havlir DV, Ignacio CC (1997). Recovery of replication-competent HIV despite prolonged suppression of plasma viremia.. Science.

[4] Finzi D, Hermankova M, Pierson T, Carruth LM, Buck C (1997). Identification of a reservoir for HIV-1 in patients on highly active antiretroviral therapy.. Science.

[5] Chun TW, Nickle DC, Justement JS, Large D, Semerjian A (2005). HIV-infected individuals receiving effective antiviral therapy for extended periods of time continually replenish their viral reservoir.. J Clin Invest.

[6] Chun TW, Nickle DC, Justement JS, Meyers JH, Roby G (2008). Persistence of HIV in gut-associated lymphoid tissue despite long-term antiretroviral therapy.. Journal of Infectious Diseases.

[7] Murray JM, Emery S, Kelleher AD, Law M, Chen J (2007). Antiretroviral therapy with the integrase inhibitor raltegravir alters decay kinetics of HIV, significantly reducing the second phase.. Aids.

[8] Steigbigel RT, Cooper DA, Kumar PN, Eron JE, Schechter M (2008). Raltegravir with optimized background therapy for resistant HIV-1 infection.. N Engl J Med.

[9] Buzon MJ, Codoner FM, Frost SD, Pou C, Puertas MC (2011). Deep Molecular Characterization of HIV-1 Dynamics under Suppressive HAART.. PLoS Pathog.

[10] Hazuda DJ, Anthony NJ, Gomez RP, Jolly SM, Wai JS (2004). A naphthyridine carboxamide provides evidence for discordant resistance between mechanistically identical inhibitors of HIV-1 integrase.. Proceedings of the National Academy of Sciences of the United States of America.

[11] Goffinet C, Allespach I, Oberbremer L, Golden PL, Foster SA (2009). Pharmacovirological impact of an integrase inhibitor on human immunodeficiency virus type 1 cDNA species in vivo.. J Virol.

[12] Buzon MJ, Massanella M, Llibre JM, Esteve A, Dahl V (2010). HIV-1 replication and immune dynamics are affected by raltegravir intensification of HAART-suppressed subjects.. Nature Medicine.

[13] Boyum A (1968). Separation of leukocytes from blood and bone marrow. Introduction.. Scand J Clin Lab Invest Suppl.

[14] Suzuki K, Shijuuku T, Fukamachi T, Zaunders J, Guillemin G (2005). Prolonged transcriptional silencing and CpG methylation induced by siRNAs targeted to the HIV-1 promoter region.. J RNAi Gene Silencing.

[15] Cordery DV, Hesse K, Amin J, Cooper DA (2010). Raltegravir and unboosted atazanavir dual therapy in virologically suppressed antiretroviral treatment-experienced HIV patients.. Antiviral Therapy.

[16] Delaugerre C, Charreau I, Braun J, Nere ML, de Castro N (2010). Time course of total HIV-1 DNA and 2-long-terminal repeat circles in patients with controlled plasma viremia switching to a raltegravir-containing regimen.. Aids.

[17] Yukl SA, Shergill AK, McQuaid K, Gianella S, Lampiris H (2010). Effect of raltegravir-containing intensification on HIV burden and T-cell activation in multiple gut sites of HIV-positive adults on suppressive antiretroviral therapy.. Aids.

[18] Yukl SA, Gianella S, Sinclair E, Epling L, Li Q (2010). Differences in HIV burden and immune activation within the gut of HIV-positive patients receiving suppressive antiretroviral therapy.. Journal of Infectious Diseases.

[19] Chun TW, Justement JS, Murray D, Hallahan CW, Maenza J (2010). Rebound of plasma viremia following cessation of antiretroviral therapy despite profoundly low levels of HIV reservoir: implications for eradication.. Aids.

[20] Guadalupe M, Sankaran S, George MD, Reay E, Verhoeven D (2006). Viral suppression and immune restoration in the gastrointestinal mucosa of human immunodeficiency virus type 1-infected patients initiating therapy during primary or chronic infection.. J Virol.

[21] Poles MA, Boscardin WJ, Elliott J, Taing P, Fuerst MM (2006). Lack of decay of HIV-1 in gut-associated lymphoid tissue reservoirs in maximally suppressed individuals.. J Acquir Immune Defic Syndr.

[22] Ruiz L, van Lunzen J, Arno A, Stellbrink HJ, Schneider C (1999). Protease inhibitor-containing regimens compared with nucleoside analogues alone in the suppression of persistent HIV-1 replication in lymphoid tissue.. Aids.

[23] Shen L, Peterson S, Sedaghat AR, McMahon MA, Callender M (2008). Dose-response curve slope sets class-specific limits on inhibitory potential of anti-HIV drugs.. Nature Medicine.

[24] Kieffer TL, Finucane MM, Nettles RE, Quinn TC, Broman KW (2004). Genotypic analysis of HIV-1 drug resistance at the limit of detection: virus production without evolution in treated adults with undetectable HIV loads.. Journal of Infectious Diseases.

[25] Joos B, Fischer M, Kuster H, Pillai SK, Wong JK (2008). HIV rebounds from latently infected cells, rather than from continuing low-level replication.. Proceedings of the National Academy of Sciences of the United States of America.

[26] Dinoso JB, Kim SY, Wiegand AM, Palmer SE, Gange SJ (2009). Treatment intensification does not reduce residual HIV-1 viremia in patients on highly active antiretroviral therapy.. Proceedings of the National Academy of Sciences of the United States of America.

[27] McMahon D, Jones J, Wiegand A, Gange SJ, Kearney M (2010). Short-course raltegravir intensification does not reduce persistent low-level viremia in patients with HIV-1 suppression during receipt of combination antiretroviral therapy.. Clinical Infectious Diseases.

[28] Grant PM, Palmer S, Bendavid E, Talbot A, Slamowitz DC (2009). Switch from enfuvirtide to raltegravir in virologically suppressed HIV-1 infected patients: effects on level of residual viremia and quality of life.. J Clin Virol.

[29] Gandhi RT, Zheng L, Bosch RJ, Chan ES, Margolis DM (2010). The effect of raltegravir intensification on low-level residual viremia in HIV-infected patients on antiretroviral therapy: a randomized controlled trial.. PLoS Med.

[30] Svarovskaia ES, Barr R, Zhang X, Pais GC, Marchand C (2004). Azido-containing diketo acid derivatives inhibit human immunodeficiency virus type 1 integrase in vivo and influence the frequency of deletions at two-long-terminal-repeat-circle junctions.. J Virol.

[31] Hazuda DJ, Felock P, Witmer M, Wolfe A, Stillmock K (2000). Inhibitors of strand transfer that prevent integration and inhibit HIV-1 replication in cells.. Science.

[32] Zhu W, Jiao Y, Lei R, Hua W, Wang R (2011). Rapid Turnover of 2-LTR HIV-1 DNA during Early Stage of Highly Active Antiretroviral Therapy.. PLoS ONE [Electronic Resource].

[33] Koelsch KK, Boesecke C, McBride K, Gelgor L, Danta M (2011). HIV-1 RNA and DNA decay characteristics during treatment with raltegravir in antiretroviral naive patients.

